# Pediatric Integrative Medicine in Residency (PIMR): Description of a New Online Educational Curriculum

**DOI:** 10.3390/children2010098

**Published:** 2015-03-17

**Authors:** Hilary McClafferty, Sally Dodds, Audrey J. Brooks, Michelle G. Brenner, Melanie L. Brown, Paige Frazer, John D. Mark, Joy A. Weydert, Graciela M. G. Wilcox, Patricia Lebensohn, Victoria Maizes

**Affiliations:** 1 Arizona Center for Integrative Medicine, University of Arizona College of Medicine, Tucson, AZ 85724, USA; E-Mail: hmcclafferty@email.arizona.edu; 2 Psychiatry and Medicine, University of Arizona College of Medicine, Tucson, AZ 85724, USA; E-Mail: sdodds1@email.arizona.edu; 3 Arizona Center for Integrative Medicine, University of Arizona College of Medicine, Tucson, AZ 85724, USA; E-Mail: brooksaj@email.arizona.edu; 4 Eastern Virginia Medical School, Children’s Hospital of the King’s Daughters, Norfolk, VA 23507, USA; E-Mail: Michelle.Brenner@CHKD.org; 5 Department of Pediatrics, University of Chicago Comer Children’s Hospital, Chicago, IL 60637, USA; E-Mail: mbrown@peds.bsd.uchicago.edu; 6 Community Faculty, Eastern Virginia Medical School, Children’s Hospital of the King’s Daughters, Norfolk, VA 23507, USA; E-Mail: jpaigefrazer@icloud.com; 7 Pediatric Pulmonary Medicine, Lucile Packard Children’s Hospital, Stanford University School of Medicine, Palo Alto, CA 94305, USA; E-Mail: jmark@stanford.edu; 8 Department of Pediatrics, University of Kansas Medical Center, Kansas City, KS 66160, USA; E-Mail: jweydert@kumc.edu; 9 Department of Pediatrics, University of Arizona, Tucson, AZ 85724, USA; E-Mail: gwilcox@peds.arizona.edu; 10 Arizona Center for Integrative Medicine, University of Arizona College of Medicine, Tucson, AZ 85724, USA; E-Mail: plebenso@email.arizona.edu; 11 Arizona Center for Integrative Medicine, University of Arizona College of Medicine, Tucson, AZ 85724, USA; E-Mail: vmaizes@email.arizona.edu

**Keywords:** integrative medicine, pediatric integrative medicine, residency education, online education, complementary medicine

## Abstract

Use of integrative medicine (IM) is prevalent in children, yet availability of training opportunities is limited. The Pediatric Integrative Medicine in Residency (PIMR) program was designed to address this training gap. The PIMR program is a 100-hour online educational curriculum, modeled on the successful Integrative Medicine in Residency program in family medicine. Preliminary data on site characteristics, resident experience with and interest in IM, and residents’ self-assessments of perceived knowledge and skills in IM are presented. The embedded multimodal evaluation is described. Less than one-third of residents had IM coursework in medical school or personal experience with IM. Yet most (66%) were interested in learning IM, and 71% were interested in applying IM after graduation. Less than half of the residents endorsed pre-existing IM knowledge/skills. Average score on IM medical knowledge exam was 51%. Sites endorsed 1–8 of 11 site characteristics, with most (80%) indicating they had an IM practitioner onsite and IM trained faculty. Preliminary results indicate that the PIMR online curriculum targets identified knowledge gaps. Residents had minimal prior IM exposure, yet expressed strong interest in IM education. PIMR training site surveys identified both strengths and areas needing further development to support successful PIMR program implementation.

## 1. Introduction

Integrative medicine (IM) is prevention-based medicine that emphasizes the therapeutic patient-clinician relationship and uses all appropriate therapies [1]. IM has unique potential in pediatrics, where acquisition of healthy habits may confer lifelong benefits.

The integrative approach is personalized and addresses nutrition, mind-body medicine, sleep, exercise, whole medical systems (e.g., traditional Chinese medicine), environmental health, and social support.

Interest in IM is significant, driven by consumer demand for care that is cost effective and better aligned with patient values [2,3]. A 2005 Institute of Medicine statement recommended that health professional schools include education on complementary medicine at all training levels [4], highlighting the need for physician education. Guidelines on IM education have been published for medical students and family medicine residents [5,6,7]. Fellowship competencies exist for IM [8], and Board certification is now available for physicians [9].

Training in IM occurred quickly in some specialties. A four-year combined residency and fellowship in family medicine and integrative medicine launched in 2004 [10]. A 200-hour online IM curriculum (Integrative Medicine in Residency, IMR) developed in 2008 [11] is now used by more than 900 residents and 50 faculty members at 42 residencies.

Pediatrics lacks IM training programs, despite data from the 2007 National Health Interview Survey demonstrating that nearly 12% of all children used complementary therapies, with prevalence increasing to 50% in those with chronic illnesses [12]. Pediatricians’ desire for education about complementary therapies was documented in an American Academy of Pediatrics (AAP) members’ survey (n = 733) [2,13].

Despite significant IM use in children and interest among pediatricians, only 16 of 143 pediatric academic programs surveyed reported having IM programs in 2012 [3]. This gap presented an opportunity to design, implement, and evaluate a pilot program (Pediatric Integrative Medicine in Residency, PIMR). Alignment of the curriculum with newly developed Accreditation Council for Graduate Medical Education (ACGME) core competencies facilitated introduction of material on empathy, self-regulation skills, and the importance of self-care in residency training [14].

This article describes the PIMR program, a 100-hour online curriculum developed at the Arizona Center for Integrative Medicine (AzCIM) at the University of Arizona, currently being implemented at five residencies. Successful implementation will rely on a program’s capacity to adopt new curriculum, and on resident readiness to learn about IM. Therefore, data will be presented on residency site characteristics, resident experience with and interest in IM, and residents’ self-assessments of perceived IM knowledge and skills.

## 2. Methods

### 2.1. Curriculum Development

Content development was based on guidelines from the joint ACGME and American Board of Pediatrics (ABP) “Pediatric Milestone Project” [15], competencies in IM [8], literature review of pediatric IM topics, and input from nationally recognized pediatric faculty. The curriculum provides: (1) an introduction to pediatric IM; (2) a review of foundational topics; and (3) case-based IM management of common conditions. Content was piloted at the University of Arizona pediatric residency then refined based on faculty and resident feedback and a needs assessment questionnaire distributed to faculty and residents at two academic pediatric training programs. Refinements included emphasis on self-care, case-based learning, and intake and treatment planning. Authors were fellowship-trained integrative pediatricians. Table 1 summarizes the curriculum.

The online curriculum is modular. Self-contained units can be adapted for use in core rotations, used within electives, or distributed longitudinally. Interactivity is facilitated with case-based teaching. Onsite teaching and experiential activities tailored by the faculty site leaders round out the program. Onsite activities may include case conferences, self-care assessments, and experiential seminars. An annual faculty retreat provides faculty support and maintenance of current IM training.

The PIMR program’s website is the hub of the resident learning community and includes online dialogues for questions and comments. Faculty site leaders track resident participation and course completion through an online dashboard. Faculty resources are housed on the PIMR website and include an article archive, intake forms, handouts, and patient education materials. Linked access is provided to the Natural Medicines Database.

Evaluation of the PIMR program’s curriculum is embedded into the website, organized into four components: (1) medical knowledge test and self-assessment; (2) course completion rates; (3) curriculum evaluation; and, (4) assessment of resident wellbeing and wellness behaviors. Both quantitative and qualitative methods are used. The University of Arizona Institutional Review Board (IRB) approved the study, as did required pilot site IRBs.

**Table 1:** Pediatric Integrative Medicine in Residency (PIMR) curriculum content.

Core Content	No. Hours
Introduction to Integrative Medicine	3
Introduction to Integrative Medicine	1
Medical Informatics	2
Self-Care	7
Introduction to Self-Care	1
Burnout & Stress	1
Mindfulness in Medicine	1
The Anti-Inflammatory Diet	2
Exercise & Sleep	1
Self-Care Wrap-up	1
Mind-Body	14
Introduction to Integrative Mental Health	6
Spirituality & Health Care	2
Mind-Body Medicine in Practice	6
Nutrition & Physical Activity	12
Nutrition Fundamentals	6
Nutrition Case Studies	5
Physical Activity for Children	1
Dietary Supplements	19
Micronutrients & Supplements: An Introduction	1
Vitamins & Minerals	7.5
Common Dietary Supplements	6.5
Botanical Foundations	4
Whole Systems	13
Whole Systems Introduction	5
Manual Medicine	8
Clinical Focus	32
Motivational Interviewing	3
Integrative Intake & Treatment Plan	3
Integrative Pediatric Neurology	5
Environmental Medicine: An Integrative Approach	6
Immunizations	1
Integrative Dermatology	4
Integrative Respiratory Health	10
TOTAL HOURS	100

### 2.2. Pilot Sites and Selection

In 2012, five residency programs were selected to participate in a three-year project to implement and evaluate the PIMR program’s curriculum. Site inclusion criteria included endorsement by the Department Chair and Residency Director, and agreement to deliver the entire 100-hour curriculum to all residents over three years. Pilot sites include four university affiliated or based residencies and one community hospital residency 16. Sites vary in annual class size from 9 to 28 residents.

### 2.3. Measures

The following measures were used to assess program capacity to implement the curriculum and resident interest and readiness to learn about IM:

***A. Site Capacity to Implement the PIMR Program: Pilot Site Characteristics*.** Within the first year of the PIMR program’s implementation, program leaders at each site completed an 11-item checklist assessing infrastructure characteristics supportive of PIMR implementation. Characteristics include an established IM culture, faculty background in IM, and site activities supporting the PIMR curriculum.

***B. Resident Interest in and Readiness for IM Training*.** Resident interest in and readiness for learning IM was assessed with three measures: (1) An eight-item ***post-match survey*** assessing prior IM/CAM (complementary alternative medicine) medical school coursework, personal use of IM/CAM, interest in learning IM, and interest in applying IM upon graduation. Items concerning prior IM experience were rated dichotomously (yes/no). Interest in learning and applying IM were rated on a 5-point scale; (2) A 32-item ***resident self-assessment questionnaire*** measuring confidence with IM knowledge and practice skills and ability to provide an IM approach to specific medical conditions. Items were rated on a 5-point scale from strongly disagree to strongly agree; and, (3) A 49-item ***medical knowledge test*** based on course content.

## 3. Results and Discussion

### 3.1. A. Site Capacity to Implement the PIMR Program: Pilot Site Characteristics

Four of five sites had the two most commonly found site readiness characteristics: an IM practitioner working onsite (IM culture), and faculty with IM training and resources. Sites varied in overall capacity to implement the PIMR program, ranging from endorsing only 1 characteristic (site E) to endorsing 8 (of the possible 11) characteristics (site C). See Table 2.

### 3.2. B. Resident Interest in and Readiness for IM Training

*Sample*. Of the 107 incoming class of 2016 residents, 97 completed the post-match, self-assessment, and/or medical knowledge measures (91% response rate). Of these, 75% were female, 68% were Caucasian, and 5% were Hispanic. Most were US medical school graduates (77%), 7% were osteopathic medical school graduates, and 16% were foreign medical school graduates.

*Post-match Survey*. The survey was completed prior to the July 1 start date. Less than one-third had IM/CAM coursework in medical school or personal experience with IM/CAM modalities. Sixty-six percent were interested in learning IM and 71% indicated interest in applying IM after graduation. See Table 3.

**Table 2:** Site characteristics by PIMR pilot site—baseline assessment.

Site	Site A	Site B	Site C	Site D	Site E	# Sites	% Sites
Faculty practicing IM consultation in the residency			X	X		2	40%
IM consultation available on site			X	X		2	40%
Other practitioners working on site	X	X	X	X		4	80%
MD and DO accredited residency, with osteopathic manipulation teaching on site		X				1	20%
IM fellowship available			X		X	2	40%
IM Culture Site Total	1	2	4	3	1	N/A	N/A
Faculty leader fellowship trained?	X	X	X	X		4	80%
Faculty leader with designated time to work on IM teaching	X	X	X	X		4	80%
Faculty Characteristics Site Total	2	2	2	2	0	N/A	N/A
Other IM teaching, rotation (1 month, 1–2 weeks), IM electives			X	X		2	40%
Case conferences monthly						0	0%
IM Retreats						0	0%
Support for residents applying knowledge in the clinic			X	X		2	40%
Additional IM Activities Site Total	0	0	2	2	0	N/A	N/A

**Table 3:** Postmatch survey results—N = 95 responding.

Item	N	Percent	Mean ±SD
*IM/CAM Experience*			
Required IM/CAM coursework in medical school—N/% Yes		28.7%	N/A
IM/CAM electives in medical school—N/% Yes		25.5%	N/A
Personal Use of IM/CAM therapies or visits with IM/CAM providers—N/% Yes		30.9%	N/A
*Interest in IM in Residency/Practice*			
Interest in learning IM in residency—N/% Interested/Very Interested		66.0%	3.7 ± 1.2
Interest in applying IM in practice after residency—N/% Interested/Very Interested		71.3%	3.7 ± 1.2

*Self-Assessment.* The highest rated knowledge/skills items concerned: patient behavior change, familiarity with diets targeting cardiovascular disease, and physical activity recommendations. The lowest rated knowledge/skills items concerned dietary supplements and whole systems medicine. The ability to apply an integrative approach to medical conditions was highest for sleep, depression, and migraines, and lowest for rheumatoid arthritis, hyperlipidemia, and Type II Diabetes. See Table 4.

**Table 4:** Self-assessment of IM knowledge and skills/applying IM approach to medical conditions—means, standard deviations, and percent endorsing *(Items are presented from highest to lowest mean rating)* N = 91.

Item	Mean	SD	% Agree/Strongly Agree
Knowledge/Skills			
I know how to assess a patient's readiness to change behavior.	3.31	0.77	48.6%
I know how to facilitate a patient's efforts at changing behaviors.	3.22	0.75	45.0%
I know the fundamental components of the Mediterranean and DASH diets as they relate to reduce risk of cardiovascular disease.	3.12	1.03	40.4%
I am aware of the different physical activity recommendations for children and adolescents.	3.03	0.84	38.5%
I can identify areas in my clinical setting that could be enhanced to promote wellbeing and healing.	2.93	1.07	35.8%
I know what the fundamental tenets of Integrative Medicine are.	2.56	0.86	16.5%
I feel competent in identifying nutritional needs based on gender, age and special populations for health promotion and disease prevention.	2.56	0.84	19.3%
I know how to take a spiritual history.	2.41	0.85	14.7%
I know the science of different mind-body techniques such as meditation, mindfulness, guided imagery, and biofeedback.	2.34	0.91	12.8%
I can identify the similarities and differences among the manual medicine modalities of massage, physical therapy, osteopathy and chiropractic.	2.33	0.97	14.7%
I can make recommendations in a patient-centered manner about an integrative treatment plan.	2.27	0.82	11.0%
I can identify the different components of an integrative treatment plan.	2.24	0.77	9.2%
I can identify the elements of an Integrative Patient intake.	2.23	0.79	7.3%
I know how to engage patients to assess mind-body interactions and their effects on health and wellness.	2.22	0.83	10.1%
I know the different theoretical and philosophical principles of Traditional Chinese Medicine (TCM), Ayurvedic Medicine, homeopathy, and Naturopathy.	1.91	0.92	7.3%
I know how to interpret the labels on herbal medicines.	1.89	0.83	4.6%
I can identify authoritative sources about botanicals.	1.87	0.80	4.6%
I know how to recommend botanicals to patients appropriately and safely.	1.67	0.67	0.9%
Medical Conditions			
Sleep disorders	2.70	1.04	28.4%
Depression	2.69	1.05	26.9%
Migraine Headaches	2.54	1.02	26.6%
Obesity	2.47	0.93	19.4%
Allergies	2.43	0.94	20.2%
Menstrual disorders	2.38	0.98	15.6%
ADHD	2.37	0.97	20.4%
Irritable Bowel Syndrome	2.37	1.01	15.6%
Asthma	2.34	0.90	12.8%
Eating Disorder	2.32	0.90	11.0%
Metabolic Syndrome	2.31	0.91	12.8%
Diabetes Mellitus type II	2.30	0.91	13.8%
Hyperlipidemia	2.30	0.91	13.8%
Rheumatoid arthritis	2.19	0.86	9.2%

*Medical Knowledge*. The average medical knowledge score was 51.3%, ranging from 35% to 78% (n = 76).

## 4. Discussion

Integrative medicine offers a powerful approach to a healthy lifestyle and can expand treatment options in children and adolescents. Use of complementary therapies is high in children, especially those living with chronic illnesses [12]. Pediatricians desire education about pediatric integrative medicine, yet few educational resources exist. The PIMR program was designed to fill this educational gap. Embedded into conventional training, it prepares pediatric residents to better serve the needs of their patients.

Preliminary results indicate that the PIMR curriculum targets identified knowledge gaps. The self-assessment and medical knowledge measures confirm the need for residents to receive exposure to this information. For example, few had awareness of IM approaches to common pediatric diagnoses such as asthma, attention deficit hyperactivity disorder, or migraine headaches. Self-assessment items specific to IM, such as knowledge of dietary supplements, received lower ratings, while more conventional topics, e.g., patient readiness to change, received higher ratings. Less than one-third had prior education or personal experience in IM. While deficits in skills and knowledge in IM would be expected upon starting residency, administration of the self-assessment and medical knowledge measures annually will allow us to track growing mastery of the curriculum content and identify content areas needing refinement.

Program implementation relies on the ability of residency programs to deliver IM education and to create a culture supportive of IM. Our initial site survey identified faculty background in IM and presence of affiliated IM practitioners as strengths across the sites. Further development is needed to support onsite IM educational activities. Site characteristics will be assessed annually to track evolution of the PIMR program’s implementation and to identify characteristics associated with curriculum completion. Evaluation of the IMR program in family medicine to date indicates that the best predictors of successful program implementation are requiring program completion for graduation and including a greater number of onsite IM activities [11].

## 5. Conclusions

Success of the PIMR program will likely depend on residents’ openness, interest, and readiness for IM training. In our survey, two-thirds of the residents expressed an interest in IM, and almost three-fourths were interested in applying IM after graduation. These findings, coupled with the self-assessment and medical knowledge results, suggest pediatric residents are interested, yet unschooled in IM when entering residency. The PIMR program provides a flexible, online curriculum that may satisfy resident interest and fill this knowledge gap. Evaluation of the curriculum is ongoing, and content will be refined in subsequent years to address identified learning gaps and feedback by participating residents and faculty.
